# Mediation of Endogenous *β*-Endorphin in the Plasma Glucose-Lowering Action of Herbal Products Observed in Type 1-Like Diabetic Rats

**DOI:** 10.1093/ecam/nen078

**Published:** 2010-10-19

**Authors:** I. M. Liu, J. T. Cheng

**Affiliations:** ^1^Department of Pharmacy, Tajen University, Yen-Pou, Ping Tung Shien, Taiwan; ^2^Department of Pharmacology, College of Medicine, National Cheng Kung University, Tainan City, Taiwan

## Abstract

Recently, there have been advances in the development of new substances effective in managing diabetic disorders. Opioid receptors couple multiple systems to result in various biological effects, although opioids are best known for analgesia. In the present review, we used our recent data to describe the advance in plasma glucose-lowering action of herbal products, especially the mediation of *β*-endorphin in glucose homeostasis of insulin-deficient diabetes. In type 1-like streptozotocin-induced diabetic rats, we identified many products purified from herbs that show a dose-dependent plasma glucose-lowering action. Increase in *β*-endorphin secretion from the adrenal gland may activate peripheral opioid *μ*-receptors (MOR) to enhance the expression of muscle glucose transporters and/or to reduce hepatic gluconeogenesis at the gene level, thereby leading to improved glucose utilization in peripheral tissues for amelioration of severe hyperglycemia. It has also been observed that stimulation of *α*_1_-adrenoceptors (*α*_1_-ARs) in the adrenal gland by some herbal products is responsible for the increase in *β*-endorphin secretion via a phospholipase C-protein kinase dependent pathway. However, an increase in *β*-endorphin secretion from the adrenal gland by herbal products can function via another receptor. New insights into the mediation of endogenous *β*-endorphin activation of peripheral MOR by herbal products for regulation of glucose homeostasis without the presence of insulin have been established. Therefore, an increase in *β*-endorphin secretion and/or direct stimulation of peripheral MOR via an insulin-independent action might serve as the potential target for development of a therapeutic agent or promising adjuvant in intensive plasma glucose control.

## 1. Introduction

Diabetes mellitus, which ranks among the top 10 causes of mortality in the world, is a metabolic disease characterized by abnormally high levels of glucose in the blood and/or urine. This metabolic disorder often leads to disability from vascular complications, including coronary artery disease, cerebrovascular disorder, renal failure, blindness and limb amputation, in addition to neurologic complications and premature death [1]. In order to normalize the plasma glucose level, dietary restrictions, exercise and oral glucose-lowering agents are widely used [2].

A widely recommended approach for the control of hyperglycemia is to administer a parenteral insulin preparation that provides a constant addition of circulating insulin for 12–24 h to augment or replace the deficient endogenous insulin secretion [3, 4], although insulin resistance is initially recognized in insulin-treated patients. Like other hormone-sensitive pathways, the intracellular pathway of insulin action is continuously being regulated by multiple influences. Continuous exposure to insulin causes a reduction in the number of receptors on the cell surface by promoting internalization as well as degradation of hormone-occupied receptors [5]. The insulin receptor is linked with tyrosine kinase that activates itself and then transmits its stimulatory message after the phosphorylation of selected tyrosine. During continuous exposure to insulin, the kinase activity of the receptor is diminished as a result of combined effects of various factors [6–8]. Also, chronic exposure of the cell to insulin may result in a diminished concentration of downstream elements, including insulin receptor substrate proteins [8]. The mechanisms underlying the concentration- and time-dependent induction of insulin resistance are complex and incompletely defined. Thus, it is imperative to provide new targets achieving equal and/or superior effects on insulin to control glycemia with less insulin resistance.

Opioid receptors are coupled to multiple systems and play a role in various biological effects, including analgesia, miosis, bradycardia, general sedation, hypothermia, insensitivity and depression of flexor reflexes [9]. Opiods have a well-known role in lowering pain [10], in addition to modulation of the immune system and endocrine processes [11]. The peripheral effect of opiates on glucose homeostasis through pancreatic islet hormone secretion has also been documented [12, 13]. The opioid peptide, *β*-endorphin, has been shown to regulate insulin, glucagon, and somatostatin release from isolated Langerhans islets [14–16]. In addition to the finding that *β*-endorphin can inhibit glucose production in conscious dogs, *β*-endorphin has an ability to increase insulin secretion via activation of opioid receptors located in pancreatic *β*-cells [17, 18]. From the effect of opioids on glucose homeostasis, opioids or opioid receptor activation may have merit in glycemic control. However, the effect of opioids on glucose metabolism in the absence of insulin remains obscure.

Recently, we provided a new evidence that activation of peripheral opioid *μ*-receptors (MOR) might modify glucose metabolism-associated genes to improve glucose utilization and reduce hepatic gluconeogenesis, thus leading to a decrease in high plasma glucose in streptozotocin-induced diabetic rats (STZ-diabetic rats). The deficient function of pancreatic *β*-cells in STZ-diabetic rats has been documented and rats with STZ-induced diabetes are widely used as an animal model of type 1-like diabetes [19]. The effect of opioids on glucose homeostasis in type 1-like diabetic rats may in fact be produced by mechanisms other than insulin. Currently, there is an enormous increase in the application of herbal and other alternative medicines for the treatment of diabetic disorders. Thus, chemical compounds or herbal products with the ability to enhance *β*-endorphin secretion and/or peripheral MOR activation, but not an opiate-like central nervous effect, might be valuable as a therapeutic intervention or an attractive adjuvant for diabetic disorders. Herein, we describe recent advances in insulin-independent mechanism(s) of herbal products in the management of glucose metabolism in insulin-deficient diabetes.

## 2. Endogenous *β*-Endorphin Participates in the Plasma Glucose-Lowering Action in an Insulin-Deficient State

### 2.1. Roles of Endogenous Opioids on Glucose Homeostasis

Previous studies have demonstrated that different forms of stress activate the endogenous opioid system, which in turn contributes to the changes in systemic cardiovascular function and endocrine responses occurring under these conditions [20–22]. Exposure to a cold environment may increase sympathetic nervous activity, inducing an elevation of plasma norepinephrine (NE) and result in hyperglycemia [23]. However, in STZ-diabetic rats, the plasma glucose-lowering response is observed after cold exposure at 4°C for 1 h with an elevation of *β*-endorphin-like immnoreactivity (BER) in plasma, which does not occur in normal rats [24]. The decrease in plasma glucose and increase of BER in response to cold stress are blocked by prazosin at a dose sufficient to abolish *α*_1_-adrenoceptors (ARs) activity. Also, the lowering of plasma glucose in STZ-diabetic rats by cold stress is abolished in the presence of a naloxone dose sufficient to block opioid receptors. Opioid receptor activation thus appears to be related to the plasma glucose-lowering action of cold stress in STZ-diabetic rats.

Although many physiologic actions of endogenous *β*-endorphin are recognized to be mediated by MOR, including the regulation of plasma glucose [25, 26], mediation of opioid *δ*-receptors (located on skeletal muscles) in the hypoglycemic effect of BER has been demonstrated [27–29]. We used MOR knockout mice to verify that MOR are involved in the regulation of plasma glucose in the absence of insulin [30]; the plasma glucose-lowering response to cold stress was not present in STZ-diabetic mice lacking MOR. Also, exogenous *β*-endorphin induced plasma glucose-lowering activity was abolished in MOR knockout STZ-diabetic mice [30]. Therefore, activation of MOR appears to be responsible for the plasma glucose-lowering response to cold stress in insulin-deficient diabetic animals.

### 2.2. Activation of *α*-ARs in the Adrenal Medulla Enhance *β*-Endorphin Secretion in an Insulin-Deficient State

Although *β*-endorphin is released along with adrenocorticotrophic hormone from the pituitary gland [31], the presence of endogenous opioids in the adrenal medulla has been identified in several species [32–35]. Various forms of polypeptides that cross-react with opioid antisera are stored together with catecholamines and soluble proteins in the granules of the adrenal gland. Bilateral adrenalectomy abolishes the plasma glucose-lowering response to cold stress in type 1-like diabetic animals [30]. Therefore, activation of MOR by opioids from the adrenal gland appears to be responsible for the plasma glucose-lowering response to cold stress in diabetic animals with insulin deficiency.

The binding site of *α*_1_-ARs in theadrenal medulla has been observed [36]. We have demonstrated that pharmacologic manipulation with methoxamine, an agonist of *α*_1_-ARs, reduced plasma glucose along with an elevation of plasma BER in STZ-diabetic rats, but a similar elevation of plasma BER by methoxamine has not been observed in normal rats [24]. A dose-dependent increase in plasma NE and *β*-endorphin has been demonstrated in STZ-diabetic rats receiving prostaglandin (PG)E_2_ injections, and these PGE_2_-induced actions are blocked by prazosin at a dose sufficient to abolish *α*_1_-ARs effects [37]. We also observed that phenylephrine enhances BER secretion from isolated adrenal medulla of Wistar rats in a concentration-dependent manner and this action is abolished by prazosin [38]. Secretion of *β*-endorphin from the adrenal glands of insulin-deficient hyperglycemic rats in response to *α*_1_-ARs activation is further supported by the finding that the plasma glucose-lowering action of PGE_2_ is deleted in STZ-diabetic rats undergoing bilateral adrenalectomy and no increase in plasma *β*-endorphin results in these rats even at highest doses of PGE_2_ [37]. Activation of *α*_1_-ARs in the adrenal medulla for the regulation of endogenous opioid secretion should thus be considered. Indeed, our data support that pituitary gland-independent release of endogenous opioids is exists in peripheral organs [39–41].

### 2.3. Signals for the Increase of *β*-Endorphin Secretion after Activation of *α*-ARs in the Adrenal Medulla

It has been established that *α*_1_-ARs belong to the family of G protein-coupled receptors which initiate signals by activating phospholipase C (PLC)-dependent hydrolysis of phisphatidylinositol 4,5 biphosphate [41, 42]. This enzyme may generate the second messengers, inositol-1,4,5-triphosphate (which releases Ca^2+^ from intracellular stores) and diacylglycerol (DAG; which synergizes with Ca^2+^ to activate protein kinase C (PKC) [41, 42]. U73122, an aminosteroid derivative, has widely been used as a specific inhibitor of PLC [43]. After comparison with its negative control, U73343 [44], the response sensitive to this inhibitor can be identified as an activation of membrane-bound PLC. It has been demonstrated that U73122 abolishes phenylephrine-stimulated BER secretion from the rat adrenal medulla, but U73343, the negative control, failed to modify this action of phenylephrine [38]. The role of PKC in the increase of BER secretion by *α*_1_-ARs in the rat adrenal medulla has also been characterized by using chelerythrine [45] and GF 109203X [46] because both compounds inhibit PKC in a competitive manner with respect to the phosphate acceptor. Indeed, the action of phenylephrine to stimulate BER secretion from isolated rat adrenal medulla is attenuated by chelerythrine or GF 109203X in a concentration-dependent manner. This survey showed the involvement of the PLC-PKC pathway in the activation of *α*_1_-ARs related to the higher secretion of *β*-endorphin from the adrenal gland.

Multiple subtypes of *α*_1_-ARs have been characterized and the receptor is classified into three native subtypes (*α*_1A_, *α*_1B_ and *α*_1D_) with corresponding cloned counterparts (*α*_1a_, *α*_1b_ and *α*_1d_) [47–51]. It has been demonstrated that phenylephrine-stimulated *β*-endorphin secretion in isolated rat adrenal medulla is attenuated by pretreatment with tamsulosin [38]. Although tamsulosin acts on both *α*_1A_-AR and *α*_1D_-AR, this compound is more selective for the *α*_1A_-subtype [52]. Thus, activation of *α*_1_-ARs, especially the *α*_1A_-subtype, on the adrenal medulla of insulin-deficient animals, may enhance the secretion of endogenous *β*-endorphin to regulate plasma glucose homeostasis. However, the subtype of *α*_1_-ARs presenting in the adrenal medulla has not been clearly identified and requires clarification.

### 2.4. Higher *β*-Endorphin Biosynthesis in the Adrenal Gland under an Insulin-Deficient State

Pro-opiomelanocortin (POMC), the precursor to *β*-endorphin and several other peptides, is synthesized by the following two groups of central neurons: (i) the arcuate nucleus and adjacent regions of the medial basal hypothalamus and (ii) the commissural subnucleus of the nucleus tractus solitarius [53]. The presence of endogenous opioids in the adrenal gland has been identified in several species [32, 34, 35, 54]. We observed that POMC gene expression is elevated, which is related to an increase of *β*-endorphin in the adrenal glands, resulting in higher plasma BER in STZ-diabetic rats as compared to normal rats [55]. Otherwise, a decrease in POMC mRNA level has been reported in the pituitary, but no change in the whole hypothalamus of diabetic rats [56]. In contrast, an increase in POMC mRNA level in the thymus is evident in STZ-diabetic rats, while gene expression of POMC in the spleen is unaltered [57]. This may be due to tissue difference. Nevertheless, an increase of *β*-endorphin synthesis in insulin-deficient diabetic rats supports a higher response to activate the opioid system for lowering plasma glucose.

## 3. Peripheral MOR Activation Participates in the Plasma Glucose-Lowering Action in an Insulin-Deficient State

### 3.1. Increase of Peripheral MOR Gene Expression in an Insulin-Deficient State

In our previous study, a dose-dependent lowering of plasma glucose was observed in the fasting STZ-diabetic rat 15 min after intravenous injection of exogenous *β*-endorphin; this action was abolished by pretreatment with naloxone or naloxonazine at doses sufficient to block MOR [58]. Also, agonists of MOR, including loperamide and tramadol, lower plasma glucose in STZ-diabetic rats, and this action was neutralized by MOR blockade [59, 60]. In contrast, the MOR activation-induced plasma glucose-lowering effect was not easy to obtain in animals with normal insulin action. It seems that a higher response to MOR activation might be responsible in diabetic rats lacking insulin. Many studies have indicated that diabetes or hyperglycaemia alter the sensitivity of animals to various agents [61, 62]. It has also been demonstrated that diabetic animals are more sensitive than normal controls to the hyperphagic effect of agonists specific to MOR [63]. However, diabetic animals are less sensitive than normal controls to anti-nociceptive action mediated by supraspinal MOR [64, 65]. In addition to the parameter of neuropathy in diabetic disorders [66], this may be due to the difference between the central nervous system and peripheral tissues, while little information is available regarding the change of MOR in diabetic disorders. It has been shown that the expression of *β*-endorphin action receptor on skeletal muscle is vastly increased in type 1 and type 2 diabetic animals [67, 68]. Our previous reports indicated that an increase in MOR not only existed in skeletal muscle, but the higher protein level of this receptor was also observed in the liver of STZ-diabetic rats [69]. Therefore, both the amount of opioid and the gene expression of MOR were raised in STZ-diabetic rats.

### 3.2. Activation of Peripheral MOR Enhances an Increase of Glucose Utilization under an Insulin-Deficient State

Using the intravenous glucose challenge test (IVGCT) that is available to characterize the ability of animals to clear glucose from the circulation, we observed that an intravenous injection of loperamide at a dose which activates MOR (17.6 *μ*g kg^−1^) significantly attenuates the increase of plasma glucose induced by IVGCT in STZ-diabetic rats [59]. Activation of peripheral MOR may increase glucose utilization to ameliorate hyperglycemia under the absence of insulin should be considered.

Glucose uptake or transportation depending on insulin-stimulated translocation of glucose carriers to the cell membrane belongs to the rate-limiting step in carbohydrate metabolism of skeletal muscle, a major site for glucose disposal [70]. Under basal conditions, the rate of glucose uptake into skeletal muscle is low and insulin-stimulated glucose disposal is believed to be the major regulation of plasma glucose concentration [70]. Hyperglycemia is an abnormal metabolic state characterized by a marked insulin defect in muscle [71]. We demonstrated that *β*-endorphin causes an increase in glucose uptake in isolated soleus muscles of STZ-diabetic rats to lower plasma glucose and this effect is antagonized by MOR-specific blockers [58]. The stimulatory effects of loperamide and tramadol on glucose uptake have also been shown in soleus muscles isolated from STZ-diabetic rats and the effects are blocked by an inhibitor specific to MOR [60, 72]. Thus, activation of peripheral MOR results in an increase in glucose uptake into skeletal muscle and is related to the lowering of plasma glucose under an insulin-deficient state.

The liver is also responsible for the regulation of blood glucose through its ability to store glucose as glycogen and/or to produce glucose from glycogen breakdown or gluconeogenic precursors [73]. In diabetes, elevation of blood glucose is a consequence of increased hepatic glucose output together with reduced peripheral glucose utilization [74]. We have observed that both naloxone and naloxonazine, at concentrations sufficient to block MOR, inhibit the increase in glycogen synthesis in STZ-diabetic rats [58, 60, 72]. Therefore, an increase in glucose utilization through peripheral MOR activation can be considered as one of the mechanism(s) for regulation of plasma glucose in the absence of insulin.

### 3.3. Signals for the Increase in Glucose Uptake by Peripheral MOR Activation

Opioid receptor activation couples a number of intracellular signaling pathways, including the mediation of PLC and PKC activation [75]. The uptake of radioactive glucose into isolated soleus muscle induced by *β*-endorphin is abolished by U73312, the specific inhibitor of PLC, while it is not affected by U73343, the negative control of U73312 [58]. Moreover, chelerythrine and GF 109203X diminished the stimulatory effect of *β*-endorphin on radioactive glucose uptake into isolated soleus muscle at a concentration sufficient to inhibit PKC [58]. The linkage of MOR and the PLC-PKC pathway in the regulation of glucose uptake is further characterized using the blockade of loperamide-stimulated 2-DG uptake in C_2_C_12_ cells by the inhibitors specific for PLC or PKC [76]. The data suggest that activation of MOR may increase glucose uptake in peripheral tissues via the PLC-PKC pathway to lower plasma glucose in diabetic rats lacking insulin.

A family of glucose transporters (GLUT) mediates glucose transport across the cell membrane, while the subtype 4 form (GLUT 4) is predominant in skeletal muscle [77]. Reduction in insulin-mediated glucose uptake caused by lower gene expression of GLUT 4 in diabetes has been observed [71, 78]. It has been reported that PKC is involved in the rate-limiting step in GLUT gene expression [79]. The gene expression of GLUT 4 in soleus muscles of STZ-diabetic rats is increased by loperamide after repeated injection for 3 days [72]. Activation of MOR by tramadol to increase glucose uptake into isolated soleus muscles with an elevation of GLUT 4 gene expression in STZ-diabetic rats has also been observed [60]. Therefore, the PLC-PKC pathway is linked to peripheral MOR activation for regulation of muscle GLUT 4 gene expression.

### 3.4. Decrease of Hepatic Gluconeogenesis by Peripheral MOR Activation in Insulin-Deficient State

Phosphoenolpyruvate carboxykinase (PEPCK; EC 4.1.1.32), the main catalyzing enzyme in gluconeogenesis, has widely been studied in hepatic carbohydrate metabolism [80, 81]. Studies in diabetic animals have shown that augmented gluconeogenesis is a major factor in the increase in plasma glucose that appears in fasting and post-absorptive states [74]. Downregulation of the PEPCK gene might be associated with a decrease in hepatic gluconeogenesis to result in the lowering of plasma glucose. We observed that loperamide at the dose which is effective in activating MOR, decreased the plasma glucose of STZ-diabetic rats accompanied by a marked reduction of PEPCK gene expression in the liver [72]. Similarly, an increased expression of the hepatic PEPCK gene in STZ-diabetic rats is reversed by tramadol [60]. Thus, peripheral MOR activation might act as a negative regulator to influence hepatic PEPCK gene expression and ameliorate the severe hyperglycemia in animals with insulin insufficiency. Gene expression of hepatic PEPCK is regulated by a number of hormones [81, 82], but signals for insulin to inhibit PEPCK expression are not transmitted through the PKC pathway [82]. Nevertheless, the decline in hepatic gluconeogenesis for lowering of plasma glucose mediated by peripheral MOR activation in the absence of insulin has been established.

## 4. Mediation of Endogenous *β*-Endorphin in the Plasma Glucose-Lowering Action of Herbal Products

### 4.1. Herbal Products Directly Activate *α*A-ARs on Adrenal Glands to Increase *β*-Endorphin Secretion

Modern medicine is believed to be the best treatment approach for acute conditions. However, Traditional Chinese medicine (TCM) is based on natural plants and such comprehensive and flexible treatment strategies always bring about fantastic treatment results. Recently, for the treatment of chronic conditions, most modern people prefer green and safe medicine and this tendency is becoming more and more prominent. Thus, people in the world are interested in TCM and it is gradually becoming mainstream medicine. Currently, there is an enormous increase in the application of herbs and other alternative medicines for the treatment of diabetic disorders. However, the mechanisms for these treatments remain unclear. Based on our previous studies, here, we indicated that some herbal products possess the plasma glucose-lowering property mediated by *β*-endorphin in the absence of insulin.

**Figure 1:**
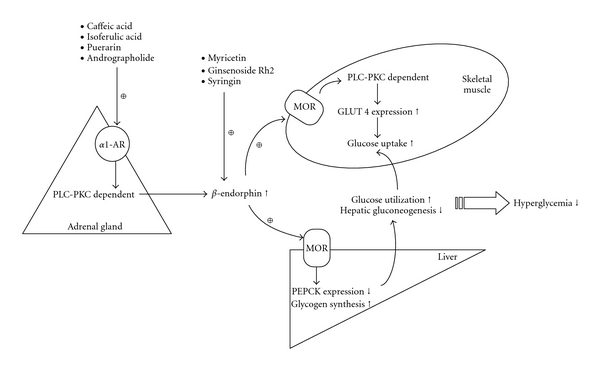
The possible mechanisms of herbal products on the plasma glucose-lowering action are mediated by activation of adrenal *α*_1_-ARs and/or stimulation of peripheral MOR in the absence of insulin. Activation of *α*1-ARs to increase *β*-endorphin release from adrenal gland was involved in the insulin-independent plasma glucose-lowering action of caffeic acid, isoferulic acid, puerarin and andrographolide. The receptor mediated on the adrenal *β*-endorphin secretion induced by myricetin, ginsenoside Rh2 and syringin is not clear, but the insulin-independent plasma glucose-lowering activity of these compounds were induced by activation of peripheral MOR via released *β*-endorphin.

Hydroxycinnamic acids, such as caffeic acid (3,4-dihydroxycinnamic acid), ferulic, acid (4-hydroxy-3-methoxycinnamic acid) and isoferulic acid (3-hydroxy-4-methoxycinnamic acid) (Table 1) are present in a large variety of fruits and vegetables, including blueberries, grapes, apples, cereal bran, broccoli, spinach and lettuce [101]. Hydroxycinnamic acid derivatives have been reported to show numerous biological activities, such as antioxidant activity, suppression of interleukin-8 production, interaction with oxytocin, inhibition of 5-lipoxygenase, effect on the arachidonic acid cascade, and anti-inflammatory activity [102–105]. The potential effect of hydroxycinnamic acid derivatives on glucose metabolism in insulin-deficient rats has also been demonstrated; activation of *α*_1A_-ARs in adrenal medulla by caffeic acid [83–85] and isoferulic acid [86–89] enhance the secretion of *β*-endorphin from the adrenal glands of STZ-diabetic rats (Table 1, Figure 1). Thus, hydroxycinnamic acid derivatives might serve as adjuvants for amelioration of high plasma glucose in patients with diabetes.

Traditionally, puerarin, a naturally occurring isoflavone C-glycoside, is used to reduce febrile symptoms, dilate arterial coronary and cerebral vessels, and decrease myocardial consumption of oxygen [106–109]. In addition, andrographolide belongs to the diterpene lactones, and has been reported to have multiple pharmacologic properties, such as protozoacidal activity, inhibition of platelet aggregation, inhibition of protein convertases-1 and -7, and furin, stimulation of cell differentiation, and anti-hepatotoxic activity, and may be developed as the drug for treatment of tissue injury, septic shock, and autoimmune diseases [110–115]. We found that puerarin [90–92] and andrographolide [93–95] also posses the ability to activate *α*_1_-ARs, especially the *α*_1A_-subtype, to enhance *β*-endorphin secretion from adrenal glands of STZ-diabetic rats (Table 1, Figure 1). Thus, these compounds possess plasma glucose-lowering property without insulin.

### 4.2. Other Herbal Products with the Ability to Increase *β*-Endorphin Secretion

An increase in *β*-endorphin secretion by herbal products does not depend on activation of *α*_1_-ARs only. The plasma glucose-lowering action of myricetin, a naturally occurring flavonoid, is commonly found in tea, berries, fruits, vegetables and the medicinal herb, mediated by activation of *α*_1_-ARs to increase the release of *β*-endorphin in type 1-like diabetic rats is not be defined [96, 97]. Although mediation of *β*-endorphin by ginsenoside Rh2, one of the ginsenosides contained in *Panax ginseng* root, or by syringin of *Eleutherococcus senticosus* to lower plasma glucose in STZ-diabetic rats have been demonstrated, the mediated receptors of the compounds remain to be identified [98–100]. Therefore, the insulin-independent plasma glucose-lowering action of herbal products is not simple and needs more investigations.

## 5. Conclusions

Taken together, the findings of our study provide new insight into the roles of both *α*_1_-ARs and MOR in glucose homeostasis in insulin-deficient diabetes. In the adrenal gland, *α*_1_-ARs activation may increase *β*-endorphin secretion via the PLC-PKC pathway, which in turn activates peripheral MOR to modify gene expression associated with glucose metabolism, including muscle GLUT 4 and hepatic PEPCK, thereby leading to improved peripheral glucose utilization and decreased hepatic gluconeogenesis for amelioration of severe hyperglycemia in type 1-like diabetes (Figure 1). Thus, chemical compounds or herbal products that could enhance *β*-endorphin secretion and/or stimulate peripheral MOR might serve as a potential agent or an attractive adjuvant for targeting plasma glucose control without insulin. However, this result seems related to the absence of insulin and is different with the changes in normal animals. Also, mediation of other opioid peptides in the activation of peripheral MOR for plasma glucose-lowering actions in diabetic animal cannot be ruled out.

## Figures and Tables

**Table 1:** Herbal products exert plasma glucose-lowering action via *β*-endorphin secretion or activation of peripheral MOR in type 1-like diabetic animals

Herbal products	Chemical names	Insulin-independent plasma glucose-lowering mechanisms
Caffeic acid	3,4-Dihydroxycinnamic acid [83–85]	Activation of *α*1-ARs on adrenal glands to increase *β*-endorphin secretion; the released *β*-endorphin then stimulates peripheral MOR leading to enhance glucose uptake and attenuate hepatic gluconeogenesis
Isoferulic acid	3-Hydroxy-4-methoxycinnamic acid [86–89]
Puerarin	4′,7-Dihydroxy-8-C-glucosylisoflavone [90–92]
Andrographolide	2.4.5.7-Trihydroxyflavone [93–95]
Myricetin	3,3′,4′,5,5′,7-Hexahydroxyflavone [96, 97]	The insulin-independent plasma glucose-lowering action of these compounds was induced by activation of peripheral MOR via released *β*-endorphin, while the receptor mediated on the enhancement of adrenal *β*-endorphin secretion is still unclear.
Ginsenoside Rh2	Proto-panaxadiol-3-o-*β*-d-glucopyranoside [98]
Syringin	4-(3-hydroxy-1-propenyl)-2,6-dimethoxyphenyl [99, 100]
